# Takotsubo Syndrome: A Pathway through the Pituitary Disease

**DOI:** 10.1155/2016/9219018

**Published:** 2016-02-25

**Authors:** Rui Plácido, Ana Filipa Martins, Susana Robalo Martins, Sónia do Vale, Ana G. Almeida, Fausto Pinto, João Martin Martins

**Affiliations:** ^1^Hospital Santa Maria, Cardiology Department, Lisbon Academic Medical Centre, CCUL, Lisbon, Portugal; ^2^Hospital de Santa Maria, Avenida Professor Egas Moniz, 1649-035 Lisboa, Portugal

## Abstract

Takotsubo cardiomyopathy (TTC) is characterized by reversible left ventricular apical and/or midventricular hypokinesia with unknown etiology. The clinical presentation is similar to acute myocardial infarction in the absence of significant obstructive coronary artery disease. Various predisposing factors have been related to TTC, such as acute neurological illnesses, endocrine diseases, pain, and emotional stress. We present the first description of an association between TTC cardiomyopathy and panhypopituitarism. This case reinforces the connection between the hormonal and cardiovascular systems. Furthermore, it supports the importance of a comprehensive and integrated medical history in the approach of a patient with cardiac disease, towards clinical decision-making.

## 1. Introduction

Takotsubo cardiomyopathy (TTC) is a novel cardiomyopathy that has been firstly described by Sato et al. [1] as an important consideration in the differential diagnosis of acute coronary syndrome. The presenting features of TTC are similar to those of myocardial ischemia after acute plaque rupture, but the characteristic distinctions are regional wall motion abnormalities that extend beyond a single coronary vascular bed and the absence of epicardial coronary occlusion. A preceding emotional or physical stressor is common [2]. The presentation can include life-threatening symptoms and hemodynamic compromise. Recently a substantial rate of death and complications after the acute phase of TTC was shown, with a long-term follow-up revealing a significant rate of death from any cause (5.6% per patient-year) and a rate of major adverse cardiac and cerebrovascular events (9.9% per patient-year) [2].

Marked increased levels of catecholamines and metanephrines are found in the acute phase and they probably account for a possible acute coronary spasm and/or focal myocardial dysfunction with contraction band necrosis/myocytolysis underlying the clinical condition [3]. Since the prognosis is much better and a different pharmacologic approach is required, angiotensin-converting–enzyme inhibitors or angiotensin receptor blockers are preferred [2], while inotropes should be avoided; the differential diagnosis is crucial [4]. Little is known about individual susceptibility to TTC. However increased sympathetic autonomic activity might be casually related. More rarely it may be related to a diseased hypothalamic-pituitary-adrenal axis (HPA), probably requiring chronic increased compensatory hyperactivity of the sympathetic autonomic system [5]. We report a clinical case supporting that association.

## 2. Case Presentation

A 74-year-old man presented at the emergency department with chest pain, palpitations, and progressive dyspnea within the last 24 hours. He had a past history of high blood pressure and dyslipidemia and was being treated with lisinopril/hydrochlorothiazide and simvastatin; an acquired atrioventricular block was managed with DDDR pacing. In the last year, fatigue, asthenia, adynamia, cold intolerance, loss of muscular strength, anorexia, decreased libido, and loss of male hair pattern distribution were also reported, with blood pressure decreasing whereat lisinopril/hydrochlorothiazide was interrupted. On physical examination, blood pressure was 102/84 mmHg, heart rate 65 beats per minute, respiratory rate 28 breaths per minute, and normal oxygen saturation (ambient air) and temperature 36.6°C. He had signs of heart failure (jugular venous distention, peripheral edema, and pulmonary congestion) and cardiac auscultation was remarkable for a protodiastolic gallop.

**Figure 1:**
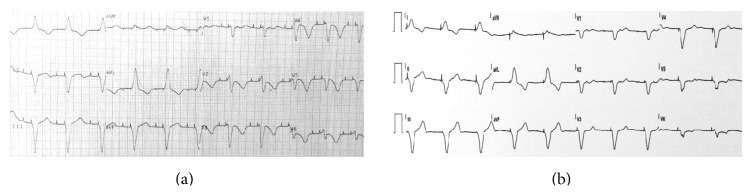
(a) Initial electrocardiogram showing an atrioventricular sequential paced rhythm with left bundle branch block morphology complexes, no ST-segment deviation, deeply inverted T waves on DI, aVL and precordial leads, and a prolonged QTc interval (560 ms). (b) Electrocardiogram 3 months after discharge.

**Figure 2:**
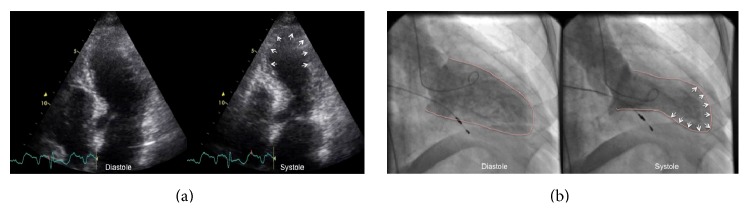
(a) Transthoracic echocardiography (apical four-chamber view) during the initial admission, demonstrating apical ballooning (white arrows). (b) Left ventriculography images in diastole and systole, showing typical type of takotsubo cardiomyopathy with apical ballooning, dyskinesia (white arrows), and left ventricle basal hypercontractility.

Electrocardiogram showed an atrioventricular sequential paced rhythm with left bundle branch block morphology complexes, no ST-segment deviation, deeply inverted T waves on DI, aVL and precordial leads, and a prolonged QTc interval (560 ms) (Figure 1(a)). Complete blood count, serum electrolytes, renal function parameters, and glycaemia were normal. Troponin I (<0.07 ng/mL) was mildly elevated (0.79 ng/mL). Echocardiography showed a nondilated left ventricle with apical dyskinesia and hyperdynamic basal contraction assuming a systolic ballooning pattern, and a moderately depressed (37%) ejection fraction (Figure 2(a); Video 1 in Supplementary Material available online at http://dx.doi.org/10.1155/2016/9219018).

Acute coronary syndrome and TTC were considered in the differential diagnosis. Coronarography showing no significant coronary artery disease and a left ventriculogram revealing the typical apical ballooning (Figure 2(b); Video 2) favor the former hypothesis. Cardiac magnetic resonance was not performed given the presence of a noncompatible pacemaker.

During hospitalization the patient was managed with diuretics, beta-blocker, and angiotensin-converting enzyme inhibitor. Laboratory data showed a peak troponin I of 1,38 ng/mL. Pheochromocytoma/paraganglioma was excluded by urinary meta- and normetanephrines measurement (High Performance Liquid Chromatography) and the patient was referred for endocrine consultation.

A formal pituitary dynamic test was performed after an overnight fast: growth hormone-releasing hormone 100 *μ*g, corticotropin-releasing hormone 100 *μ*g, thyrotropin-releasing hormone 200 *μ*g, and gonadotropin-releasing hormone 100 *μ*g bolus (intravenous) at time 0; baseline/peak hormone levels were as follows: growth hormone (<2 ng/mL): 0.15/0.18; prolactin (2–18 ng/mL): 21/35; cortisol (4–23 *μ*g/dL): 1.5/4.1; follicle-stimulating hormone (1–18 U/L): 2.2/2.8; luteinizing hormone (3–35 U/L): 0.3/1.1; other baseline values were insulin-like growth factor 1 (48–188 ng/mL): 33; adrenocorticotropin (0–46 pg/mL): 31; triiodothyronine (60–181 ng/dL): 81; thyroxine (4.5–10.9 *μ*g/dL): 2.2; free thyroxine (0.89–1.80 ng/dL): 0.42; total testosterone (188–772 ng/dL): <10. All measurements were obtained with standardized chemiluminescence immunoassays; age and gender specific reference values at 8.00–10.00 h am after an overnight fast are presented within square brackets. Thyroid autoantibodies were negative.

Brain magnetic resonance imaging revealed a normal sized and positioned pituitary gland with no morphological abnormalities and a centered pituitary stalk.

A diagnosis of idiopathic panhypopituitarism was established and the patient was prescribed hydrocortisone 20 + 10 mg daily (oral), levothyroxine 100 *μ*g daily (oral), and testosterone enanthate 250 mg every fortnight (intramuscular). The usual instructions were given orally and by writing regarding stressful conditions.

After 3 months of optimized heart failure treatment the patient showed clinical improvement, normalization of left ventricular function, and reversibility of T waves changes and QT prolongation (Figure 1(b)). Interestingly, in this case, the repolarization changes were clearly interpretable notwithstanding the presence of a right ventricular paced rhythm. In that time, lost weight was recovered and the patient regained normal physical vigour without other complaints.

## 3. Discussion

This case begins with an unusual presentation of TTC. There was no record for any particular acute emotional or physical stress; the patient is male while TTC is much more common in women and the presentation is that of an acute coronary syndrome with marked left ventricular dysfunction [3, 6].

TTC is an acute, reversible dysfunction of the left ventricle in the absence of significant obstructive coronary disease. Recently, Templin et al. [2] prospectively showed that the most common pattern was the apical type (81.7%) followed by the midventricular (14.6%), the basal (2.2%), and the focal (1.5%) types. The condition is especially prevalent in postmenopausal women but the true prevalence of TTC remains uncertain because of its underrecognition. The long-term prognosis is generally favorable. The pathophysiology of TTC is not established but is likely multifactorial, involving the vascular, endocrine, and central nervous systems. Several mechanisms have been postulated to link the sympathetic hyperactivity and myocardial dysfunction including diffuse coronary artery spasm, coronary microcirculation alterations, and direct catecholamine mediated myocyte injury [3, 7]. In 70% of the documented cases there is an apparent trigger leading to catecholamine-induced myocardial injury [6]. Further investigation is needed to define the connection between these triggers and their effect on the vulnerable myocardium.

In the acute phase, TTC is sometimes indistinguishable from acute myocardial infarction with respect to clinical symptoms, ECG changes, and cardiac biomarkers such as troponin and creatine kinase. Fast and accurate diagnosis on admission remains challenging, and exclusion of significant obstructive coronary artery disease is mandatory. Thus, there is a need for sensitive and specific biomarkers for the early diagnosis of TTC. MicroRNAs are a class of highly conserved and small noncoding posttranscriptional regulators of diverse cellular processes that have been recently considered as biomarkers in cardiovascular disease [8]. Jaguszewski et al. [9] recently described a signature of circulating microRNAs for sensitive and specific identification of TTC in the acute phase. The significant upregulation of stress- and depression-related microRNAs suggested a role of central and/or peripheral nervous system in TTC.

Retrospectively the patient of our case presented complains suggesting pituitary failure—low blood pressure, cold intolerance, loss of physical vigour and motivation, decreased libido, scanty body hair—although this is a rare condition, and these symptoms are by themselves rather common in older men [7]. Endocrine evaluation unequivocally revealed complete anterior pituitary failure, but the reason is not apparent. Combined pituitary failure by itself is a very rare condition—estimated prevalence 46/100.000 and estimated annual incidence 4/100.000—and at least in adult/old patients most commonly depends on pituitary tumors [10, 11]. Other less common causes include head trauma [12], for which the patient had no recollection and besides pituitary stalk section generally occurs; hemochromatosis and other metabolic conditions, for which no evidence was found in this case (iron, transferrin, and ferritin levels were normal and the pituitary was not enlarged); vascular, that would be likely in this age group, namely, subarachnoid, hemorrhage [13]. However, there was no evidence for pituitary apoplexy at the CT scan. In fact most other conditions leading to combined pituitary failure, namely, infections, inflammatory, granulomatous, immune, or neoplastic disorders, generally present with structural abnormalities in the pituitary gland that can be shown by MRI [7, 10, 11]. Of course a congenital or genetic and functional defect is mostly unlikely, given patient age and clear evidence for previous normal growth and development, including two fathered children. In short, unequivocal combined pituitary failure is present but the reason is unclear. This occurs in around 10–20% of the cases, and an etiology sometimes is apparent only many years later [14]. An acute and transient defect is also unlikely since the patient had previous evidence of pituitary failure and maintained that condition for as long as two years of follow-up requiring substitutive therapy. Also, the possibility of a neuroendocrine adaptation to an acute/chronic condition may be discarded. That adaptation, for which the nonthyroidal patient syndrome is the best-known pattern, is associated with increased cortisol levels [15]. By the same token, a drug effect is unlikely.

Besides some perplexing features, a most interesting point can be made that progressive multiple pituitary failure may require an increased sustained sympathetic activity to cope with daily life physical and emotional challenges, since it is by itself certainly a stressful condition. Curiously enough, to our knowledge, other reports of TTC and endocrine disease always resolve around the theme of pheochromocytoma [16] or failure of the HPA axis [5, 17, 18]; to our knowledge, this is the first report of the association of TTC with adult onset multiple pituitary hormonal failure. Chronic activation of the sympathetic nervous system may on the other hand favor the development of TTC, as has been suggested by some reports regarding behavioral medicine [3]. Regarding combined pituitary failure, increased sympathetic drive may be particularly deleterious, since androgens may downregulate the stress response and may present direct vasodilator effects on coronary arteries [19], and low testosterone predicts the development of atherosclerosis and cardiovascular events [20] while glucocorticoids may play a supportive role on myocardial function [21] and hypothyroidism may impair coronary blood flow [22].

TTC represents an acute heart failure syndrome that is associated with a substantial risk for adverse events. The great variety of triggering factors in this disease needs to be illuminated and new investigations are required. Furthermore, a heightened vigilance in the treatment of these patients is crucial to improve prognosis.

In a way, the gap is narrowing between “Voodoo death” [23] as described by Sir Cannon in the 40s and TTC reported in the 90s. It remains to be seen if this association of combined pituitary failure and TTC is also reported by other authors. TTC by itself was also a long present but unknown condition until Sato first formally described it.

## Supplementary Material

Video 1: Two-dimensional transthoracic echocardiography, apical four-chamber view showing a non-dilated left ventricle with apical dyskinesia and hyperdynamic basal contraction assuming a systolic ballooning pattern, with a moderately depressed ejection fraction.
Video 2: Left ventriculography showing the typical apical ballooning, with apical dyskinesia and hyperdynamic basal contraction.

## References

[1] Sato H., Tateishi H., Uchida T., Kodama K., Haze K., Hon M. (1990). Takotsubo type cardiomyopathy due to multivessel spasm. *Clinical Aspect of Myocardial Injury: From Ischemia to Heart Failure*.

[2] Templin C., Ghadri J. R., Diekmann J. (2015). Clinical features and outcomes of takotsubo (stress) cardiomyopathy. *The New England Journal of Medicine*.

[3] Wittstein I. S., Thiemann D. R., Lima J. A. C. (2005). Neurohumoral features of myocardial stunning due to sudden emotional stress. *The New England Journal of Medicine*.

[4] Ghadri J. R., Ruschitzka F., Lüscher T. F., Templin C. (2014). Takotsubo cardiomyopathy: still much more to learn. *Heart*.

[5] Sakihara S., Kageyama K., Nigawara T., Kidani Y., Suda T. (2007). Ampulla (takotsubo) cardiomyopathy caused by secondary adrenal insufficiency in ACTH isolated deficiency. *Endocrine Journal*.

[6] Bybee K. A., Kara T., Prasad A. (2004). Transient left ventricular apical ballooning: a syndrome that mimics ST-segment elevation myocardial infarction. *Annals of Internal Medicine*.

[7] Burt M. G., Ho K. K. Y., Jameson J. L., De Groot L. J., Kretser D. M. (2016). Hypopituitarism and growth hormone deficiency. *Endocrinology. Adult and Pediatric*.

[8] Gupta S. K., Bang C., Thum T. (2010). Circulating MicroRNAs as biomarkers and potential paracrine mediators of cardiovascular disease. *Circulation: Cardiovascular Genetics*.

[9] Jaguszewski M., Osipova J., Ghadri J.-R. (2014). A signature of circulating microRNAs differentiates takotsubo cardiomyopathy from acute myocardial infarction. *European Heart Journal*.

[10] Regal M., Páramo C., Sierra J. M., Garci-Mayor R. V. (2001). Prevalence and incidence of hypopituitarism in an adult Caucasian population in northwestern Spain. *Clinical Endocrinology*.

[11] van Aken M. O., Lamberts S. W. J. (2005). Diagnosis and treatment of hypopituitarism: an update. *Pituitary*.

[12] Richmond E., Rogol A. D. (2014). Traumatic brain injury: endocrine consequences in children and adults. *Endocrine*.

[13] Kreitschmann-Andermahr I. (2005). Subarachnoid hemorrhage as a cause of hypopituitarism. *Pituitary*.

[14] Nyström H. F., Saveanu A., Barbosa E. J. L. (2011). Detection of genetic hypopituitarism in an adult population of idiopathic pituitary insufficiency patients with growth hormone deficiency. *Pituitary*.

[15] Van den Berghe G., Jameson J. L., De Groot L. J., Kretser D. M. (2016). Endocrine aspects of critical care medicine. *Endocrinology. Adult and Pediatric*.

[16] Lassnig E., Weber T., Auer J., Nömeyer R., Eber B. (2009). Pheochromocytoma crisis presenting with shock and tako-tsubo-like cardiomyopathy. *International Journal of Cardiology*.

[17] Ukita C., Miyazaki H., Toyoda N., Kosaki A., Nishikawa M., Iwasaka T. (2009). Takotsubo cardiomyopathy during acute adrenal crisis due to isolated adrenocorticotropin deficiency. *Internal Medicine*.

[18] Murakami M., Matsushita N., Arai R. (2012). Isolated adrenocorticotropin deficiency associated with delirium and takotsubo cardiomyopathy. *Case Reports in Endocrinology*.

[19] Jones R. D., English K. M., Hugh Jones T., Channer K. S. (2004). Testosterone-induced coronary vasodilatation occurs via a non-genomic mechanism: evidence of a direct calcium antagonism action. *Clinical Science*.

[20] Hyde Z., Norman P. E., Flicker L. (2012). Low free testosterone predicts mortality from cardiovascular disease but not other causes: the Health in Men study. *Journal of Clinical Endocrinology and Metabolism*.

[21] Ren R., Oakley R. H., Cruz-Topete D., Cidlowski J. A. (2012). Dual role for glucocorticoids in cardiomyocyte hypertrophy and apoptosis. *Endocrinology*.

[22] Lee S. J., Kang J. G., Ryu O. K. (2009). The relationship of thyroid hormone status with myocardial function in stress cardiomyopathy. *European Journal of Endocrinology*.

[23] Cannon W. B. (1942). ‘Voodoo’ death. *American Anthropologist*.

